# Which symptoms are linked to a delayed presentation among melanoma patients? A retrospective study

**DOI:** 10.1186/s12885-016-2978-6

**Published:** 2017-01-03

**Authors:** Sally Jane O’Shea, Zoe Rogers, Fiona Warburton, Amanda J. Ramirez, Julia A. Newton-Bishop, Lindsay J. L. Forbes

**Affiliations:** 1 Section of Epidemiology and Biostatistics, Cancer Genetics Building, Leeds Institute of Cancer and Pathology, University of Leeds, St. James’s University Hospital, Beckett Street, Leeds, LS9 7TF UK; 2 Division of Cancer Studies, King’s College London, Research Oncology, Guy’s Hospital, Bermondsey Wing, 3rd Floor, Great Maze Pond, London, SE1 9RT UK

**Keywords:** Melanoma, Delayed diagnosis, Behavioural symptoms

## Abstract

**Background:**

The incidence of melanoma is rising. Early detection is associated with a more favourable outcome. The factors that influence the timing of a patient’s presentation for medical assessment are not fully understood.

The aims of the study were to measure the nature and duration of melanoma symptoms in a group of patients diagnosed with melanoma within the preceding 18 months and to identify the symptoms and barriers associated with a delay in presentation.

**Methods:**

A questionnaire was distributed to a random sample of 200 of the 963 melanoma patients who had participated in the Cancer Patient Experience Survey 2010 and were known to be alive 1 year later. Data were collected on symptoms, duration of symptoms prior to presentation and the reasons for not attending a doctor sooner.

**Results:**

A total of 159 patients responded to the questionnaire; 74 (47%) were men; mean age was 62 (range 24–90) years. Of the 149 patients who reported a symptom, 40 (27%) had a delayed presentation (i.e. >3 months). A mole growing bigger was the most common symptom and reporting this symptom was significantly associated with a delayed presentation (odds ratio (OR) 2.04, 95% confidence interval (95% CI) 1.14–5.08). Patients aged ≥65 years were less likely to report a barrier to presentation and were less likely to delay than those under 40, although this was of borderline statistical significance (OR 0.28, 95% CI 0.08–1.00).

**Conclusions:**

This study highlights that an enlarging mole is a significant symptom influencing the timing of presentation. Increasing public awareness of the signs of melanoma and of the importance of early presentation is desirable. Health professionals should take advantage of the opportunity to educate patients on such symptoms and signs where feasible. Further exploration of the barriers to presentation in younger people should be considered.

**Electronic supplementary material:**

The online version of this article (doi:10.1186/s12885-016-2978-6) contains supplementary material, which is available to authorized users.

## Background

The incidence of melanoma is rising, with a greater than four-fold increase within the past 40 years in the United Kingdom (UK) [1]. In 2012, there were 2148 deaths from melanoma in the UK [1]. Thin, non-ulcerated melanomas (Breslow thickness <1 mm) have the best prognosis [2], suggesting that early diagnosis and treatment would contribute to better outcomes. Urgent referral to specialist care is indicated in cases of suspected melanoma and the 2-week wait system is designed to limit healthcare-associated delay. Patient factors, however, crucially influence the timing of presentation [3–11]. It has previously been shown that changing moles may not cause patient concern or even be recognised as a feature of skin cancer [12] and the reasons for delay are complex, including a lack of recognition of the symptoms as significant and not wanting to “waste” the doctor’s time [8, 10, 11, 13]. It is critical to better understand the reasons for patient delay in order to effect change and to overcome obstacles to early detection. In this study, we report the nature of symptoms prompting presentation to medical services, reported by melanoma patients participating in a UK survey of cancer patients.

## Methods

In November 2011, we sent a questionnaire (NHS Cancer Symptoms Questionnaire (Additional file 1)) to a random sample of 200 patients with melanoma (International Classification of Diseases-10 (ICD-10) code C43) [14]. These were drawn from a sample of 963 patients with melanoma who had responded to the English Department of Health’s Cancer Patient Experience Survey in mid-2010 [15], were still alive in November 2011, and had agreed that they could be recontacted.

Of these 200, one further person was subsequently reported to have died. The questionnaire asked about the nature and duration of melanoma-related symptoms prior to patient presentation at their general practitioner. Participants could tick one or more boxes in response to each question within the questionnaire, i.e. lists were provided, consisting of the most common symptoms (e.g. a mole changing shape or growing bigger), pre-defined time intervals (e.g. <2 weeks, more than 2 weeks but less than 4 weeks) and possible reasons for delay (e.g. I didn’t realise the problem or symptom was serious). Free text boxes were also included to capture any additional information that was not included in the list. Further details of the methods are available in a previous report [4].

As a survey of perceptions of National Health Service (NHS) patient care, no formal ethical approval was sought for the Cancer Patient Experience Survey by the Department of Health. However, all patients approached as part of the survey had given written consent to being recontacted.

All participants were assigned an Index of Multiple Deprivation (IMD), based on the average level of deprivation of the area where they lived. Participants were then categorised into five categories according to quintiles of IMD for all of England [16].

The duration of symptoms was calculated as the time interval between first noticing the symptom that led to diagnosis and presenting to a doctor. A delay in presentation was defined as a duration of symptoms of 3 months or more.

In order to estimate the risk of a delay in presentation, we calculated odds ratios (with 95% confidence intervals) for delay according to age (<40, 40–64, ≥65 years), gender, deprivation index category, tumour site and barriers to presentation reported. We also calculated odds ratios for delay by symptoms reported; in each analysis the reference group was the group of patients who did not report that particular symptom. It was not possible to calculate adjusted estimates due to small numbers. We also examined whether age, gender and deprivation index category were associated with reporting barriers, using χ^2^ tests.

All statistical analyses were done using STATA v12.

## Results

There was an 80% response rate (*n* = 159) to the request for completion of the second survey. Age at diagnosis was available for 145 patients. The mean age was 62 (range 24–90) years, which is slightly older than UK norms for melanoma patients. Forty-three per cent (*n* = 68) of patients were aged 65 years or older. Forty-seven per cent of participants (*n* = 74) were male, with a male-female ratio of 1:1.1, which is similar to the overall male:female ratio for melanoma in the UK [1]. Twenty-seven per cent (*n* = 43) and 7% (*n* = 11) of respondents were in the least and most deprived deprivation index categories, respectively. The ratio of those in the least deprived to most deprived categories was 3.9:1 (compared to 2.7:1 between 2006 and 2010 in England overall) [17]. Ethnicity was available for 140 patients. All described their ethnicity as White, which reflects the epidemiology of melanoma, occurring more frequently in people with paler skin types [18]. Fifteen per cent (*n* = 24) of patients had melanomas of the head and neck, 30% (*n* = 48) on the trunk, 24% (*n* = 38) on the upper limb and 28% (*n* = 44) on the lower limb. Site was not recorded in 5 cases.

Of the 159 respondents, 149 (94%) reported symptoms at the time that medical help was sought (Table 1). Ten patients did not list any symptoms, 3 of whom reported the incidental detection of ‘moles’ by health professionals during consultations for other conditions. Participants could list one or more symptoms. Of those patients reporting symptoms, 89 (60%) patients reported either a mole growing bigger, changing shape or changing colour. The most commonly reported symptom (39%) was a ‘mole growing bigger’. Of the 149 patients who reported symptoms, 76% (*n* = 113) described a change in a pre-existing mole rather than a new mole or lesion.

**Table 1:** Type of symptoms and the time interval from onset of symptoms to presentation among melanoma patients reporting symptoms

Symptom	≤4 weeks	4 weeks – 3 months	>3 months	Duration missing	Number reporting symptom (% of all patients)
	n (%)	n (%)	n (%)	n (%)	n (%)
New lump on skin or new mole ^a^	8 (34.8)	5 (21.7)	9 (39.1)	1 (4.3)	23 (15.4)
Mole changing shape, colour or growing bigger ^b^	41 (46.1)	14 (15.7)	29 (32.6)	5 (5.6)	89 (59.7)
Mole growing bigger	24 (43.1)	9 (15.5)	22 (37.9)	2 (3.5)	58 (38.9)
Mole changing colour	22 (44.9)	7 (14.3)	18 (36.7)	2 (4.1)	49 (32.9)
Mole changing shape	19 (43.2)	7 (15.9)	16 (36.4)	2 (4.6)	44 (29.5)
Mole itchy	10 (31.3)	8 (25.0)	11 (34.4)	3 (9.4)	32 (21.5)
Mole bleeding or crusting	21 (53.9)	10 (25.6)	7 (18.0)	1 (2.6)	39 (26.2)
Lump in neck, groin or armpit	3 (100.0)	0 (0.0)	0 (0.0)	0 (0.0)	3 (2.0)

Of the 159 participants, data for symptoms were available for 149 patients and data for duration of symptoms were available for 141 patients. Participants could list more than one symptom

^a^The symptom list on the questionnaire did not include ‘new mole’. Participants who ticked ‘other’ and wrote free text suggesting a new mole have been recoded within the category ‘new lump on skin’

^b^The duration of these symptoms were reported separately and together, as melanomas, particularly of the superficial spreading subtype, might display a combination of these features

The duration of symptoms was reported by 141 patients (Table 1). Of the 149 patients who reported symptoms, 26% (*n* = 39) reported a mole bleeding or crusting and of these only 18% (*n* = 7) had a presentation of more than 3 months (defined as a delayed presentation). Only 2% (*n* = 3) of patients presented with a lump in the neck, groin or armpit, all of whom presented within 4 weeks.

A delay of greater than 3 months was reported by 27% of patients (*n* = 40). Table 2 shows the factors that were associated with a delay in presentation. Patients aged 65 and over were less likely to delay, compared to patients who were less than 40, although the association was of borderline statistical significance. Gender and tumour site were not significantly related to delay, although we noted that the proportion delaying more than 3 months was highest in patients with lesions on the head and neck compared to other sites (36% versus 27%; data not shown). Deprivation index category was not significantly associated with delay (data not shown). Those patients who reported ‘a mole growing bigger’ were more likely to have a delayed presentation compared to those who didn’t report a mole growing bigger (OR 2.04, 95% CI 1.14–5.08). Those who reported a mole changing colour or a mole changing shape were also more likely to have a delayed presentation than those who did not report these symptoms, but these associations did not reach statistical significance.

**Table 2:** Factors associated with a delay in symptomatic presentation of more than 3 months (among 141 patients reporting any symptoms and with data on time to presentation available)

		N (%) delaying presentation	Odds ratio for delay	95% CI	*p*
Age (years)	<40	6/12 (50.0)	1.00		
	40–64	19/57 (33.3)	0.50	0.14–1.76	0.28
	≥65	13/60 (21.7)	0.28	0.08–1.00	0.05
Sex	Male	16/66 (24.2)	1.00		
	Female	24/75 (32.0)	1.32	0.77–2.26	0.32
Site of primary melanoma	Head and neck	8/22 (36.4)	1.00		
	Trunk	9/43 (20.9)	0.46	0.15–1.45	0.19
	Limbs	21/71 (29.6)	0.74	0.27–2.01	0.55
	Unspecified	2/5 (40.0)	1.17	0.16–8.53	0.88
New lump on skin or new mole	No	31/119 (26.1)	1.00		
	Yes	9/22 (40.9)	1.97	0.77–5.05	0.16
Mole changing shape or colour or growing bigger	No	11/57 (19.3)	1.00		
	Yes	29/84 (34.5)	2.20	0.99–4.89	0.05
Mole growing bigger	No	18/85 (21.2)	1.00		
	Yes	22/56 (39.3)	2.04	1.14–5.08	0.02
Mole changing colour	No	22/94 (23.4)	1.00		
	Yes	18/47 (38.3)	2.03	0.95–4.33	0.07
Mole changing shape	No	24/99 (24.2)	1.00		
	Yes	16/42 (38.1)	1.92	0.89–4.17	0.10
Mole itchy	No	29/112 (25.9)	1.00		
	Yes	11/29 (37.9)	1.75	0.74–4.14	0.20
Mole bleeding or crusting	No	33/103 (32.0)	1.00		
	Yes	7/38 (18.4)	0.47	0.19–1.20	0.12

The odds ratio for delay was not calculated for a lump in the neck, groin or armpit as no patients reporting this symptom had a delayed presentation. For the odds ratios by symptom, the reference group is the group of patients who did not report that particular symptom. Age at diagnosis was available for 129 of the 141 patients reported in this table

Of the 149 patients reporting symptoms, 55% (*n* = 82) reported a barrier to symptomatic presentation, and this was associated with a greater delay in presentation (OR 5.87, 95% CI 2.37–14.50; data not shown). Forty-four per cent (*n* = 66) had not realised that the symptom was serious, which was the most common barrier reported (Table 3). Other types of barrier were much less commonly reported. People aged 65 and over were less likely to report barriers to presentation than younger people (48% versus 68%, *p* = 0.02) but there were no significant differences by gender or deprivation index category (data not shown).

**Table 3:** Reported barriers to symptomatic presentation among 149 melanoma patients who reported at least one symptom

Barrier reported	n (%)
I didn’t realise the symptom was serious	66 (44.3)
I was worried about wasting the doctor’s time	13 (8.7)
I had too many other things to worry about at the time	9 (6.0)
I was too busy to make time to go to the doctor	6 (4.0)
I was too worried about what the doctor might find	5 (3.4)
I was too scared to go and see the doctor	5 (3.4)
It was difficult to make an appointment with the doctor	7 (4.7)
I was too embarrassed to go to see the doctor	1 (0.7)
I found my doctor difficult to talk to	1 (0.7)
I didn’t feel confident talking about my symptoms with the doctor	1 (0.7)
It was difficult to arrange transport to the doctor’s	0 (0.0)

Of the 149 patients reporting symptoms, 82 reported a barrier to symptomatic presentation. Patients could select more than one barrier

## Discussion

This was a mailed questionnaire study with a good response rate of 80%. Although the responders were older than the average melanoma patient within the UK population, the sample was an appropriate reflection of the sex distribution of melanoma [1]. The participants were more affluent than the UK population in general, which has been noted previously [17]. This questionnaire was part of a larger study, aiming to capture data across a broad spectrum of cancer types. The sample size, while small, was selected to assess feasibility, and to facilitate estimation of symptom prevalence in the entire group of patients with all cancers.

The most commonly reported presenting symptom was ‘a mole growing bigger’ and this was the only symptom significantly associated with delay. Although growth of a melanocytic lesion is characteristic of melanoma acknowledged by the ABCDE rule [19] (where E indicates “evolution”), it is normal for moles or melanocytic nevi to change through early life. Moles appear in childhood, grow in early adult life and then stop growing, usually at under 5 mm in diameter. It is, perhaps, understandable that patients may seek advice only when they notice another change in the lesion, such as irregularity of shape, or even a combination of changes. We would suggest that there is a need for health promoters to develop the public’s knowledge and skills in order to distinguish the natural history of moles (melanocytic naevi) from that of a melanoma, albeit a challenging task. This study highlights the importance of education about the diameter (“D” ≥6 mm) and evolution (“E”) of lesions, in particular.

Growth of moles over the age of 40 is much less common than in younger people, but thereafter it is normal to develop a variety of other harmless skin lesions, such as seborrhoeic keratoses and cherry angiomas. Therefore, such patients become accustomed to developing new lesions. The challenge for health promoters is to teach them how to distinguish benign from malignant. The proportion of patients who presented late, having reported moles changing colour and shape was similar to those delaying before reporting a mole getting bigger, at around 35 to 40%. We hypothesise that the diagnostic difficulties experienced in this regard are similar as for growth in size.

The participants in this study appeared to recognise bleeding or a lump as being more serious. A greater proportion of patients with this symptom presented within 3 months although the odds ratio for delay was not statistically significant. Bleeding is known to occur more frequently in thicker tumours [20] and appears to be a more recognisable symptom by patients [11]. In other cancers, bleeding is perceived as an alarm symptom and is associated with a more prompt presentation when the source is urinary but not rectal [4].

Twenty-seven per cent of melanoma patients in this study did not seek medical attention for at least 3 months after a symptom was first noted. This suggests that a substantial number of patients within this group had a delayed presentation. This is concerning, given that the skin, unlike many other organs of the body, is generally readily visualised. Therefore, if a changing lesion is located at a visible body site, it would be amenable to early detection, provided the significance of the symptom(s) is appreciated. In the UK, it is NHS policy that a patient referred urgently to specialist care due to suspicion of cancer, would be seen within 2 weeks of referral [21].

We had hypothesised that tumour site and gender might have influenced delay. Older men are a ‘high-risk’ group for melanomas with a poorer prognosis: they tend to present with thicker tumours, at sites that are more difficult for patients to see, such as the back [20]. Melanomas affecting the head and neck tend to be thicker and have previously been shown to be associated with delay [22] but this was not replicated in our study. Further research with a larger sample size would be needed to analyse for interactions between age and gender on the delay in presentation by symptoms. Since awareness of the need to have the symptom evaluated may be influenced by the level of education and previous attendance at a dermatology clinic, additional research is needed to evaluate these influences.

More than half of the respondents (55%) reported factors that might put them off going to a doctor and this increased the time interval to presentation. Some patients have perceived themselves to be “low-risk” [11] and have reported a lack of general malaise, which might have contributed to delay [12]. Patients have also noted that they worry about attending their general practitioner (GP) for fear of creating a “fuss” [13]. Interestingly, those patients aged 65 years and over were less likely to report barriers than their younger counterparts.

The survey was completed by participants within approximately 18 months of a diagnosis of melanoma. This interval may have contributed to recall bias and imprecision in the recall [23]. Some selection bias might also have been introduced, as study participants were selected from those patients who were known to be alive 1 year after the Cancer Patient Experience Survey.

Further, the respondents might have been more likely to have a better disease course [6]. Clinical stage was not recorded and therefore, it was not possible to assess its impact on the results. In an effort to minimize selection bias, a random sample was chosen, however, those with less aggressive tumours may have been more likely to participate.

A further limitation of the study was that, by virtue of the study design, a number of clinical and histological features which could have influenced outcome were not recorded, e.g. histological subtype. Nodular melanoma tends to follow a more aggressive clinical course than the superficial spreading subtype [24], lacking a precancerous or “*in situ*” phase, whilst desmoplastic melanomas generally carry a better prognosis, tending to present with localized disease [25, 26]. The biology of all melanomas may not be the same: nodular melanomas are also believed to progress rapidly [27]. This means that a similar interval between symptom onset and presentation could result in differences in tumour thickness related to the biological behaviour of the tumour [27]. It was not possible to determine whether or not delay was influenced by melanoma-specific features, e.g. the lesion lacking pigmentation (amelanotic) or due to differences in Breslow depth or ulceration. ICD-10 coding was used as an indicator for body site but this method of grouping included a combination of different histological subtypes within the same code. Patients with early melanoma tend to feel well and, as a result, may not heed the initial symptoms [12].

This study suggests that barriers might influence the timing of presentation in melanoma patients. Such barriers to presentation should be explored further. Psychosocial factors have previously been reported to have more influence on younger patients with melanoma [28]. Work and childcare commitments might be contributing factors, although this was not specifically explored in our study. Older patients may have reported fewer barriers because they were already attending their doctor with other health problems but future studies may clarify the reasons for this association.

## Conclusions

The commonest symptoms of cutaneous melanoma are changes in size, shape or colour. This study suggests that patients commonly take some time to identify these changes as being of concern. We hypothesise that this may result from difficulties in identifying such changes, when normal moles do change with time and a number of benign skin lesions commonly develop on the ageing skin. We would argue that promoting public awareness about the dangers of changing skin lesions is desirable. Barriers to presentation were frequently cited by participants in this study. It is crucial to achieve a better understanding of these barriers in order to optimise the timing of presentation among melanoma patients, where early detection is paramount.

## Additional file

Additional file 1: NHS Cancer Symptoms Questionnaire. This questionnaire was completed by study participants. Details of symptoms, symptom duration and barriers to presentation were recorded. (PDF 276 kb)

## Abbreviations

95% CI: 95% confidence interval
GP: General practitioner
ICD: International classification of diseases
IMD: Index of multiple deprivation
NHS: National Health Service
OR: Odds ratio
UK: United Kingdom
