# Supplementary material for: Which symptoms are linked to a delayed presentation among melanoma patients? A retrospective study
Source: BMC Cancer. 2017 Jan 3;17:5. doi: 10.1186/s12885-016-2978-6 (PMC5209829; doi:10.1186/s12885-016-2978-6)
Supplement: Additional file 1: — NHS Cancer Symptoms Questionnaire. This questionnaire was completed by study participants. Details of symptoms, symptom duration and barriers to presentation were recorded. (PDF 276 kb) [file 12885_2016_2978_MOESM1_ESM.pdf]

Cancer Symptoms Questionnaire

This questionnaire is about the symptoms you may have had before you were diagnosed with cancer. This information will help the NHS to diagnose patients faster, and improve the quality of health services.

Who should complete the questionnaire?
The questions should be answered by the person named in the letter enclosed with this questionnaire. If that person needs help to complete the questionnaire, the answers should be given from their point of view – not the point of view of the person who is helping.

Completing the questionnaire
For each question please tick clearly inside the box that is closest to your views using a black or blue pen. Don't worry if you make a mistake; simply cross out the mistake and put a tick in the correct box.

Please **do not** write your name or address anywhere on the questionnaire.

IMPORTANT INFORMATION

To make sure the information we collect is useful, we need to collect some personal details from you and to access information held about you in other NHS databases.

By completing this questionnaire you are giving your consent for the information provided to be used for the above purposes. Specifically, you are agreeing that:

- Your personal details and relevant health information can be held and used by an organisation contracted to the Department of Health to analyse the data
- Other information about you held by the Patient Demographics Service, the Secondary Users Service, Cancer Registries and other NHS databases can be held and used by an organisation contracted to the Department of Health to analyse the data
- The information you provided in the National Cancer Patient Experience Survey 2010 can be used to help analyse the information you provide in this survey
- Your personal details can be used to send you related follow-up questionnaires in the future

Your personal information will be handled securely and anonymised after analysis and before any publication. Your personal information will not be released by the Department of Health or third party organisations working on its behalf unless required by law or where there is a clear overriding public interest.

You can withdraw the information you give the NHS in this questionnaire upon request, up to the point at which data are analysed and personal details removed.

If you have any queries about the questionnaire,
please call the FREEPHONE helpline number **on 0800 783 1775**

Taking part in this survey is voluntary
Published reports will not contain any personal details

In these first few questions we would like to know about the **MAIN** health problems or symptoms that made you go to see the GP (family doctor), **or** made you go to accident and emergency, and **that led to you being diagnosed with cancer.** These **MAIN** problems or symptoms **may have gone on for some time** before you were diagnosed**.** We would like you to tell us about any other **earlier symptoms** you may have had before you were diagnosed in question **8**.

1. How were you referred to hospital **when you were diagnosed with cancer**?

1 ☐ A GP (family doctor) referred me

2 ☐ I went (or was taken) directly to the accident and emergency (casualty) department at the hospital

3 ☐ Other (please describe)

2. Please tick your **MAIN** health problems or symptoms – you can tick more than one, but please make sure these are the problems or symptoms that took you to the doctor:

1 ☐ Mole bleeding or crusting

2 ☐ Mole changing shape

3 ☐ Mole growing bigger

4 ☐ New lump on the skin

5 ☐ Mole changing colour

6 ☐ Mole got itchy

7 ☐ Lump in neck, groin or armpit

8 ☐ I had no symptoms or health problems at all ➔ **Go to question 8**

9 ☐ Other (please describe)

3. If you can remember **the date** when these **MAIN** health problems or symptoms started, please write it here:

Day	Month	Year

If you can't remember the ***exact date****, just fill in the month and / or the year.*

4. How long was it from the time you noticed these **MAIN** health problems or symptoms and first seeing a GP or hospital doctor?

1 ☐ Less than 2 weeks

2 ☐ More than 2 weeks but less than 4 weeks

3 ☐ More than 4 weeks but less than 3 months

4 ☐ More than 3 months but less than 6 months

5 ☐ More than 6 months but less than a year

6 ☐ More than a year

7 ☐ Don't know / can't remember

5. If you can remember **the date** when you first saw a GP (family doctor) **or** you went directly to accident and emergency about these **MAIN** health problems or symptoms, please write it here:

Day	Month	Year

If you can't remember the ***exact date****, just fill in the month and / or the year.*

6. If you went to see a GP (family doctor) about these **MAIN** health problems or symptoms what did the GP do? **(Tick ONE option closest to your views)**

1 ☐ The GP referred me for tests

2 ☐ The GP referred me to see a specialist at a hospital clinic

3 ☐ The GP sent me straight to hospital that same day

4 ☐ The GP started treating me for another condition

5 ☐ The GP said the symptom wasn't serious. I **wasn't told to come back** if it continued

6 ☐ The GP said the symptom wasn't serious. I **was told to come back** if it continued

7 ☐ I did not see a GP

If you would like to give further details please use the comments box on page 4

7. Did any of the following things put you off going to see a doctor about these **MAIN** health problems or symptoms? **(Tick ALL that apply)**

1 ☐ I didn't realise the problem or symptom was serious

2 ☐ I was too embarrassed to go to see the doctor

3 ☐ I was too scared to go and see the doctor

4 ☐ I was worried about wasting the doctor's time

5 ☐ I found my doctor difficult to talk to

6 ☐ It was difficult to make an appointment with the doctor

7 ☐ I was too busy to make time to go to the doctor

8 ☐ I had too many other things to worry about at the time

9 ☐ It was too difficult to arrange transport to the doctor's

10 ☐ I was too worried about what the doctor would find

11 ☐ I didn't feel confident talking about my symptoms with the doctor

12 ☐ Something else

13 ☐ I was not put off going to see a doctor

8. Looking back, did you have any earlier health problems or symptoms **that happened before those ticked in question 2,** that you **NOW** think were due to **cancer**?

1 ☐ Yes ➔ **Go to question 9**

2 ☐ No ➔ **Go to question 13**

9. If yes, what health problems or symptoms were these? **(Tick ALL that apply)**

1 ☐ Mole bleeding or crusting

2 ☐ Mole changing shape

3 ☐ Mole growing bigger

4 ☐ New lump on the skin

5 ☐ Mole changing colour

6 ☐ Mole got itchy

7 ☐ Lump in neck, groin or armpit

8 ☐ Other (please describe)

10. Which of these earlier health problems or symptoms was the **FIRST** one that you **NOW** think was due to **cancer**? **(Tick ONE only)**

1 ☐ Mole bleeding or crusting

2 ☐ Mole changing shape

3 ☐ Mole growing bigger

4 ☐ New lump on the skin

5 ☐ Mole changing colour

6 ☐ Mole got itchy

7 ☐ Lump in neck, groin or armpit

8 ☐ Other (please describe)

11. If you can remember **the date** when this **FIRST** health problem or symptom started, please write it here:

Day	Month	Year

*If you can't remember the **exact date**, just fill in the month and / or the year.*

12. How long was it from the time you noticed this **FIRST** health problem or symptom and first seeing a doctor?

1 ☐ Less than 2 weeks
2 ☐ More than 2 weeks but less than 4 weeks
3 ☐ More than 4 weeks but less than 3 months
4 ☐ More than 3 months but less than 6 months
5 ☐ More than 6 months but less than a year
6 ☐ More than a year
7 ☐ Don't know / can't remember

13. If you can remember the date when you were **diagnosed with cancer**, please write it here:

Day	Month	Year

*If you can't remember the **exact date**, just fill in the month and / or the year.*

If there is anything else you would like to tell us about how you first came to be diagnosed with cancer please do so here.
